# Fabrication and Evaluation of pH-Sensitive Chitosan-Coated Membranes for Enhanced Oil Emulsion Filtration

**DOI:** 10.3390/membranes15090252

**Published:** 2025-08-27

**Authors:** Eunseo Choi, Siyoung Byun, Sanghyun Jeong

**Affiliations:** 1 Civil and Environmental Engineering, Pusan National University, Busan 46241, Republic of Korea; bmb6262@pusan.ac.kr; 2 Institute for Environment and Energy, Pusan National University, Busan 46241, Republic of Korea; bco0306@pusan.ac.kr

**Keywords:** chitosan, membrane, oil separation, pH-responsive, wettability

## Abstract

Oil-contaminated wastewater presents a significant environmental challenge, necessitating the development of efficient and adaptable treatment technologies. In this study, a pH-responsive chitosan-coated polyethersulfone (Ch/PES) membrane was developed and systematically evaluated for oil/water separation performance under varying pH conditions. PES was chosen as the base membrane material due to its excellent chemical resistance and mechanical durability, while Ch, a biodegradable and environmentally friendly biopolymer with pH-sensitive properties, was applied as a functional surface coating. The Ch/PES membrane was successfully fabricated and characterized by Fourier-transform infrared spectroscopy and scanning electron microscopy, confirming successful surface modification and structural integrity. Additional analyses—including underwater oil contact angle measurements, porosity assessment, and cross-sectional morphological evaluation—demonstrated the membrane’s dynamic pH-responsive wettability and pore size modulation. Oil emulsion separation experiments, conducted using sodium dodecyl sulfate-stabilized emulsions, revealed that the Ch/PES membrane achieved oil removal efficiencies exceeding 97% under acidic conditions. This enhancement was attributed to increased hydrophilicity and reduced effective pore size resulting from chitosan swelling. In contrast, under alkaline conditions, the membrane exhibited greater oleophilicity and maintained a relatively stable pore structure, leading to a reduced separation efficiency of 83.8%. Compared to the unmodified PES membrane, the Ch/PES membrane demonstrated significantly improved responsiveness and adaptability to changes in pH, underscoring its potential as a versatile platform for treating oil-contaminated wastewater of varying chemistries. These findings suggest that the Ch/PES membrane offers a promising, sustainable, and efficient solution for advanced oil/water separation applications.

## 1. Introduction

Oil-containing wastewater presents a critical environmental challenge, particularly due to its detrimental impacts on aquatic ecosystems [1]. Among various forms, oil-in-water emulsions with droplet sizes below 20 μm are especially difficult to treat because of their thermodynamic stability and fine droplet dispersion [2,3,4]. As such, the development of efficient, cost-effective, and environmentally sustainable separation technologies has become a pressing research priority.

Conventional oil/water separation methods—including centrifugal separation, biodegradation, chemical demulsification, photocatalytic degradation, gas flotation, oil skimmers, incineration, and adsorption—have been widely explored [5,6,7,8,9,10]. Likewise, diverse materials such as meshes, woven and nonwoven fabrics, sponges, aerogels, and foams have shown potential in separating buoyant or gravity-driven oil [11,12,13]. However, these traditional approaches often suffer from low separation efficiency, high operational costs, complex maintenance requirements, and the generation of secondary pollutants [14,15]. To overcome these limitations, significant research has focused on the development of advanced separation technologies and novel materials that offer superior performance, particularly in the treatment of stable emulsions. The goal is to achieve high separation efficiency while maintaining economic and environmental viability.

Membrane-based filtration has emerged as one of the most promising approaches for treating oil-in-water emulsions. This technique employs semi-permeable membranes to separate oil droplets or dissolved components from water by applying a pressure gradient, enabling the passage of clean water while retaining contaminants. Membrane processes are categorized based on pore size into microfiltration (MF), ultrafiltration (UF), nanofiltration, and reverse osmosis. Among these, MF and UF—operating within pore size ranges of 0.1–5 μm and 2–100 nm, respectively—are particularly suitable for the removal of emulsified oil droplets [16,17,18]. These processes also offer advantages such as low energy consumption and reduced operating pressures. Nevertheless, conventional MF and UF membranes face considerable limitations in complex separation scenarios. A primary drawback is their fixed wettability and selectivity—permitting only one phase (either oil or water) to pass—thereby restricting their versatility in dynamically changing or bidirectional separation applications. Furthermore, their performance declines markedly when challenged with surfactant-stabilized emulsions, which exhibit smaller droplet sizes and enhanced stability. These characteristics hinder oil rejection and membrane performance. Hence, the need for next-generation membranes with improved interfacial properties, pH-responsiveness, tunable wettability, and superior adaptability to complex emulsions is imperative. Such advanced membrane systems can provide enhanced oil/water separation efficiency, even in the presence of surfactants and other stabilizing agents, while meeting the demands of sustainability and operational feasibility.

Stimuli-responsive membranes with switchable wettability have emerged as a promising innovation to address the limitations of conventional MF and UF membranes [19]. Unlike traditional membranes, which possess fixed surface properties and are limited to separating either oil or water, stimuli-responsive membranes can dynamically adjust their wettability in response to external stimuli such as pH, temperature, or ionic strength [20,21,22,23]. This tunable behavior enables selective oil or water permeability on demand, providing enhanced control and adaptability for complex separation processes. In particular, the switchable wettability significantly improves the separation efficiency of surfactant-stabilized emulsions (oil-in-water and water-in-oil), where fine droplets and interfacial stability pose serious challenges to conventional methods. By responding to environmental triggers, these membranes enable dynamic interfacial interactions, leading to high rejection rates and efficient separation even under variable water quality conditions [24,25]. Several studies exemplify this progress. For instance, tannic acid/diethylenetriamine-coated membranes exhibit reversible wettability under solvent treatment and achieve over 98% separation efficiency for both emulsion types [19]. Yuan et al. developed an electro-responsive membrane reactor with switchable wettability. The protonation of amine groups under applied voltage enabled rapid and reversible transitions between hydrophobic and hydrophilic states, achieving efficient oil–water separation with over 99% removal and concurrent catalytic degradation of pollutants [26].

Among the various stimuli-responsive systems, pH-responsive membranes have received particular attention due to their rapid responsiveness, simple operation, and wide applicability [27]. Materials such as poly(acrylic acid), polyamines, and chitosan (Ch) have been extensively explored for such applications [28,29]. Of these materials, Ch, a naturally derived, biodegradable polysaccharide obtained by deacetylation of chitin, is especially attractive due to its abundance, biocompatibility, and environmental friendliness [30]. It can be readily processed into films, fibers, and hydrogels for diverse industrial and environmental applications, including pharmaceuticals, cosmetics, and water treatment [31,32,33]. Crucially, chitosan possesses amine groups that undergo protonation under acidic conditions, inducing electrostatic repulsion, enhanced water uptake, and polymer swelling. Conversely, in alkaline conditions, deprotonation leads to contraction of the polymer matrix due to diminished electrostatic interactions. This reversible and tunable swelling behavior makes chitosan a particularly suitable candidate for stimuli-responsive membrane systems in oil/water separation [34].

This study aims to develop and evaluate a pH-responsive membrane by coating chitosan onto a polyethersulfone (PES) substrate, targeting efficient separation of oil/water emulsions under varying pH conditions. PES was chosen for its excellent mechanical and chemical stability [35]. The Ch/PES membrane was fabricated and characterized using Fourier-transform infrared spectroscopy (FTIR) and scanning electron microscopy (SEM) to confirm successful coating and structural integrity. Underwater oil contact angle (UWOA) measurements were conducted to assess pH-responsive changes in membrane wettability, while porosity and morphology were evaluated across different pH conditions. Oil emulsion separation experiments using sodium dodecyl sulfate (SDS)-stabilized emulsions were carried out to investigate the pH-responsive wettability of the Ch/PES membrane and its correlation with separation efficiency. In particular, the membrane’s adaptive behavior under acidic and alkaline conditions was examined to evaluate its effectiveness in separating emulsified oil.

## 2. Materials and Methods

### 2.1. Materials

Chitosan (Ch, low molecular weight, MW = 50–190 kDa, CAS No. 9012-76-4), glutaraldehyde (GA; Grade II, 25% in H_2_O, CAS No. 111-30-8), and acetic acid (HAc; glacial, ≥99.7%, ACS reagent, CAS No. 64-19-7) were purchased from Sigma-Aldrich Co. for the preparation of the chitosan solution. Sodium dodecyl sulfate (SDS; ≥99.0%, ACS reagent, CAS No. 151-21-3) was also obtained from Sigma-Aldrich Co. (St. Louis, MO, USA). Isopropyl alcohol (IPA; CAS No. 67-63-0) and sodium hydroxide (NaOH; beads, ≥97.0%, CAS No. 1310-73-2) were supplied by Daejung Chemicals & Metals Co., Ltd. (Siheung, Republic of Korea). Hydrochloric acid (HCl; extra pure, 35%, CAS No. 7647-01-0) was procured from Junsei Chemical Co., Ltd. (Tokyo, Japan). All reagents were used as received without further purification. Commercial polyethersulfone (PES) membranes (disk diameter: 47 mm, pore size: 0.45 μm) were obtained from GVS Filter Technology (Zola Predosa, Italy). The oil used in the emulsion separation experiments was canola oil, with specifications detailed in Table A1.

### 2.2. Fabrication of Ch/PES Membrane

**Figure 1:**
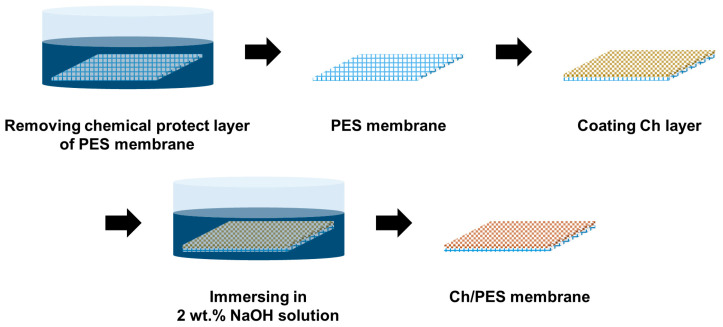
Schematic illustration of the fabrication process for the pH-responsive chitosan-coated polyethersulfone (Ch/PES) membrane.

A 1 wt.% Ch solution was prepared by dissolving Ch in a 2 wt.% HAc solution under continuous stirring at 150 rpm and 60 °C for 24 h. This concentration was selected because lower Ch contents led to insufficient coating, while higher concentrations caused severe permeability loss during separation tests. Subsequently, 25 mL of the Ch solution was mixed with 25 mL of 0.12 wt.% glutaraldehyde solution to initiate cross-linking, forming a chitosan–glutaraldehyde (Ch–GA) solution. Membrane coating was carried out using a dip-coating technique (Figure 1). Initially, PES membranes were soaked in a 30% (*v*/*v*) isopropyl alcohol (IPA) solution to remove any protective layers, then rinsed with deionized (DI) water. The cleaned membrane was fixed onto a circular reaction vessel, and the Ch–GA solution was evenly dispensed over the membrane surface. After 8 min of static coating, the excess solution was drained, and the membrane was dried in an oven at 60 °C for 24 h. To complete cross-linking and stabilize the chitosan layer, the dried membrane was immersed in a 2 wt.% sodium hydroxide (NaOH) solution for 20 min. The membrane was then thoroughly rinsed with DI water to remove residual NaOH and stored in DI water until further characterization and performance testing.

### 2.3. Characterization of Ch/PES Membranes

The chemical structure of the Ch/PES membranes was analyzed using FTIR, (Nicolet iS50, Thermo Fisher Scientific, Waltham, USA) in the spectral range of 4000–600 cm^−1^. UWOA measurements were performed using a contact angle goniometer (Phoenix-300, SEO, Gyeonggi, Republic of Korea) to evaluate the membranes’ wettability under different pH conditions.

Surface morphology and microstructure were observed using field-emission scanning electron microscopy (Supra 25, ZEISS, Oberkochen, Germany). To assess pH-responsive porosity, membranes were immersed in solutions of pH 3, 6, and 12 (adjusted with HCl or NaOH) for 30 min. The porosity was calculated based on the weight difference between the wet and dry membranes.

Membrane thickness was measured using a digital micrometer (293–334, Mitutoyo, Kawasaki, Japan). The surface zeta potential of the membranes was measured with an electrokinetic analyzer (SurPASS, Anton Paar GmbH, Graz, Austria) using a 1 mM KCl solution as the background electrolyte. The solution pH was adjusted using HCl and NaOH.

### 2.4. Oil–Water Separation Performance of Ch/PES Membrane

The oil–water separation performance was evaluated using a model oil-in-water emulsion prepared by mixing 10 mL of canola oil with 990 mL of deionized (DI) water containing 1 g of sodium dodecyl sulfate (SDS). The mixture was stirred at 1000 rpm for 30 min at room temperature. Due to the risk of instrument contamination by oil, the particle size distribution of the emulsion was analyzed using optical microscopy combined with ImageJ 1.53t software instead of a particle size analyzer.

The emulsions were adjusted to pH 3, 6, and 12 to assess the pH-responsiveness of the Ch/PES membrane. Separation experiments were performed under gravity-driven filtration without the application of pressure. The initial liquid column height was set to approximately 8 cm (~150 mL) without additional feeding during filtration. The effective membrane area was 0.00113 m^2^. The water flux (J, L m^−2^ h^−1^ or LMH) was calculated using the following equation:

J = ∆W/(A × ∆t)(1)

where ΔW is the volume of permeate (L), A is the membrane area (m^2^), and Δt is the filtration time (h). The concentration of residual oil in the permeate was quantified using a UV–visible spectrophotometer (DR6000, Hach, Loveland, CO, USA), with each measurement performed in triplicate to ensure reproducibility. Oil detection was conducted within the characteristic absorption wavelength range of 252–272 nm.

## 3. Results and Discussion

### 3.1. Characterization of Ch/PES Membrane

#### 3.1.1. Chemical Characterization of Ch/PES Membrane

**Figure 2:**
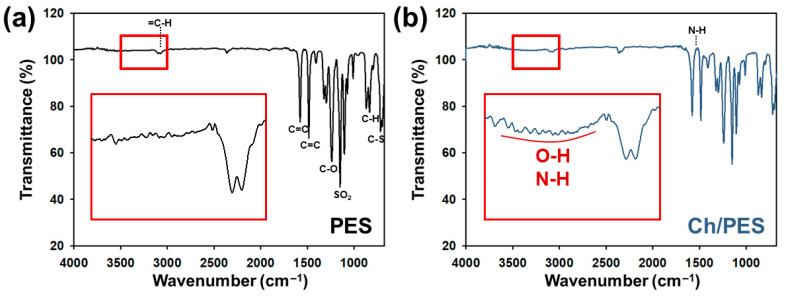
FTIR spectra of (**a**) unmodified PES and (**b**) Ch/PES membranes.

To investigate the chemical modifications introduced by coating GA-crosslinked Ch onto the PES membrane, FTIR spectroscopy was conducted. Figure 2 presents the FTIR spectra of the unmodified PES and Ch/PES membranes.

The unmodified PES membrane displayed characteristic peaks corresponding to its aromatic and sulfone functional groups. Notably, C–H stretching vibrations from the 1,4-disubstituted benzene ring appeared near 3000 cm^−1^, while C=C stretching vibrations were observed at 1585 and 1588 cm^−1^. Strong C–O stretching bands were evident in the 1275–1200 cm^−1^ region, attributed to ether linkages in the aromatic backbone. The sulfone group (–SO_2_) exhibited asymmetric and symmetric stretching vibrations in the ranges of 1250–1290 cm^−1^ and 1165–1129 cm^−1^, respectively, which are known to shift to lower frequencies when bonded to aromatic rings [35,36]. After modification with Ch, the Ch/PES membrane exhibited additional spectral features. A broad, weak absorption band centered around 3430 cm^−1^ was observed, corresponding to O–H and N–H stretching vibrations from chitosan functional groups engaged in hydrogen bonding. This peak was absent in the unmodified PES membrane, confirming successful surface modification. Furthermore, a distinct band around 1560 cm^−1^ was detected, which corresponds to N–H bending vibrations, typically associated with the amide II region, providing further evidence of chitosan immobilization and cross-linking on the PES surface [37,38,39].

#### 3.1.2. Physical Characterization of Ch/PES Membrane

**Figure 3:**
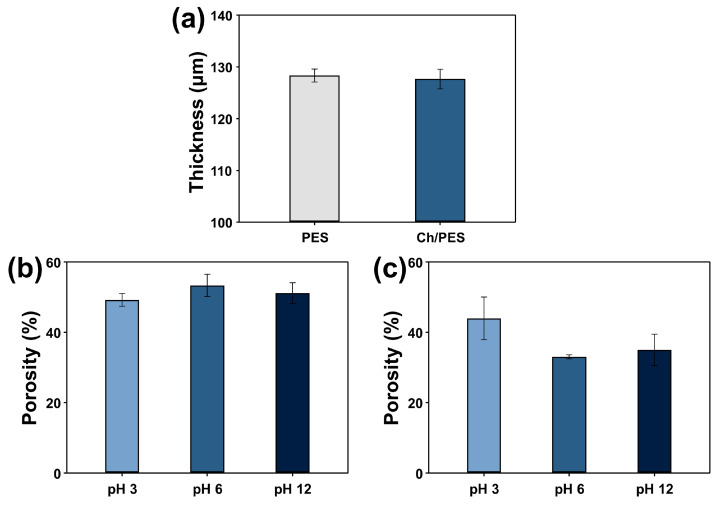
(**a**) Membrane thickness of PES and Ch/PES membranes; porosity of (**b**) PES and (**c**) Ch/PES membranes measured under varying pH conditions (pH 3, 6, and 12).

**Figure A1:**
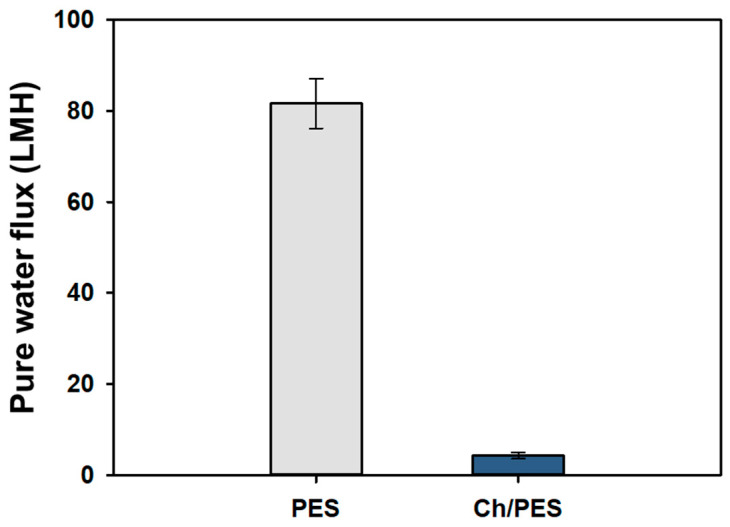
Pure water flux comparison of PES and Ch/PES membranes measured by gravity-driven filtration.

The physical properties of the PES and Ch/PES membranes were evaluated in terms of thickness, water flux, and porosity under varying pH conditions. As shown in Figure 3a, the membrane thicknesses were 128.3 μm for PES and 127.7 μm for Ch/PES, indicating that the Ch coating process did not significantly alter membrane thickness. Despite comparable thickness, a substantial difference was observed in pure water flux between the two membranes, measured via gravity-driven filtration. The pristine PES membrane exhibited a water flux of 81.57 LMH, whereas the Ch/PES membrane showed a markedly reduced flux of 4.31 LMH (Figure A1). This reduction is attributed to the hydrophilic Ch coating, which decreases effective pore size and increases resistance to water permeation.

Porosity measurements were further conducted under different pH conditions to evaluate pH-responsive behavior (Figure 3b,c). The PES membrane displayed relatively stable porosity across pH 3, 6, and 12, suggesting minimal responsiveness to environmental pH changes. In contrast, the Ch/PES membrane exhibited pronounced pH-dependent variations in porosity. Under acidic conditions, the porosity of Ch/PES increased significantly due to the swelling behavior of Ch. The protonation of amino groups in acidic environments enhances water absorption, expanding the hydrogel matrix and increasing overall porosity. Conversely, under alkaline conditions, deprotonation of amino groups reduces water uptake, resulting in lower porosity. These findings confirm the pH-responsive structural adaptability of the Ch/PES membrane, which is critical for its selective separation performance.

**Figure 4:**
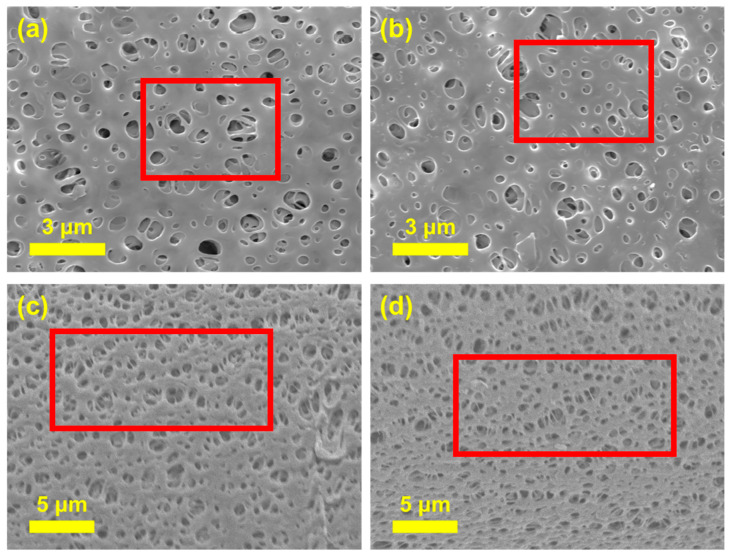
SEM images of (**a**,**b**) the top surface, and (**c**,**d**) the cross-section of PES and Ch/PES membranes at pH 6, respectively.

To examine the morphological modifications induced by the Ch coating, SEM was conducted. Figure 4a,b present the top-view SEM images of the unmodified PES and Ch/PES membranes, respectively. While both membranes exhibit a porous surface structure, the Ch/PES membrane shows a noticeable reduction in average pore size, attributed to the surface deposition of the Ch hydrogel layer. This difference is further corroborated by the cross-sectional SEM images in Figure 4c,d, which clearly reveal the denser and more compact pore architecture of the Ch/PES membrane compared to the relatively open structure of the pristine PES membrane. These results confirm that Ch coating effectively alters membrane morphology, contributing to reduced pore size and enhanced filtration selectivity.

#### 3.1.3. Swelling Behavior of Ch/PES Membrane Under Different pH Conditions

**Figure 5:**
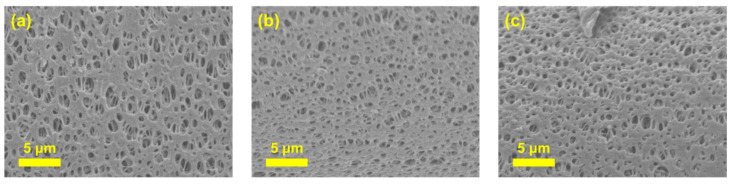
Cross-sectional SEM images of Ch/PES membranes immersed at different pH levels: (**a**) pH 3, (**b**) pH 6, and (**c**) pH 12 (Note: Figure 5b image is the same as Figure 4d image at pH 6 condition).

To assess the pH-responsive swelling behavior of the fabricated Ch/PES membranes, dried samples were immersed in aqueous solutions at pH 3, 6, and 12 for 30 min, followed by drying and cross-sectional SEM analysis. As shown in Figure 5, the membranes exhibited larger pore sizes under acidic conditions (pH 3) relative to those observed at neutral (pH 6) and alkaline (pH 12) environments. This behavior is attributed to the protonation of amino groups in Ch under acidic conditions, which induces electrostatic repulsion among polymer chains, weakening intermolecular interactions and leading to greater contraction upon drying. Conversely, at alkaline pH, the amino groups become deprotonated, diminishing electrostatic repulsion and facilitating stable polymer chain aggregation, thereby maintaining pore structure integrity after drying [40].

#### 3.1.4. Zeta Potential and Wettability of Ch/PES Membrane at Different pH Levels

**Figure 6:**
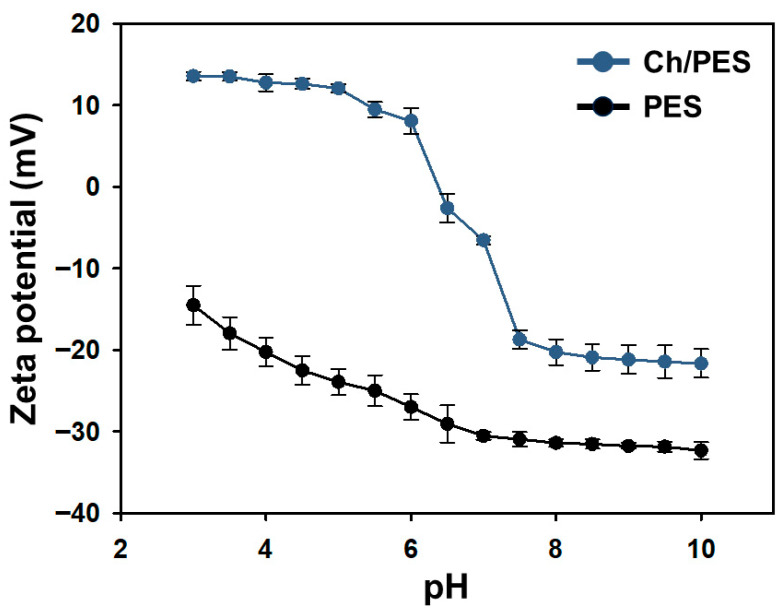
Zeta potential of PES and Ch/PES membranes measured across a range of pH values.

Figure 6 presents the zeta potential of both PES and Ch/PES membranes as a function of pH. The unmodified PES membrane consistently exhibited a negative zeta potential across the entire pH range, with values becoming increasingly negative at higher pH, indicating enhanced electrostatic repulsion of negatively charged species under alkaline conditions. In contrast, the Ch/PES membrane showed overall higher zeta potential values due to the presence of the Ch coating. Specifically, the Ch/PES membrane exhibited a positive zeta potential below pH 6, attributed to protonation of amino groups in Ch, and transitioned to a negative zeta potential above pH 6. Despite this sign reversal, the Ch/PES membrane maintained a higher absolute zeta potential than the PES membrane across all tested pH values, highlighting the significant influence of the Ch layer on the surface charge properties.

**Figure 7:**
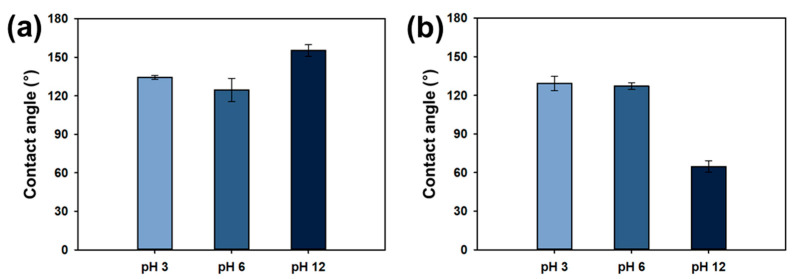
UWOA of (**a**) PES and (**b**) Ch/PES membranes at varying pH levels.

To assess the pH-dependent wettability of the Ch/PES membrane, UWOA measurements were performed. The unmodified PES membrane displayed UWOA values of 134.30 ± 5.49°, 124.51 ± 9.13°, and 155.29 ± 4.54° at pH 3, 6, and 12, respectively, indicating consistently hydrophilic behavior across all pH conditions (Figure 7a). In contrast, the Ch/PES membrane exhibited UWOA values of 129.27 ± 5.49° and 127.21 ± 2.68° under acidic and neutral conditions, respectively, confirming hydrophilicity. However, at pH 12, the UWOA sharply decreased to 64.69 ± 4.46°, indicating a transition to oleophilicity under alkaline conditions (Figure 7b).

### 3.2. Emulsified-Oil Separation Performance of the Ch/PES Membrane

**Figure 8:**
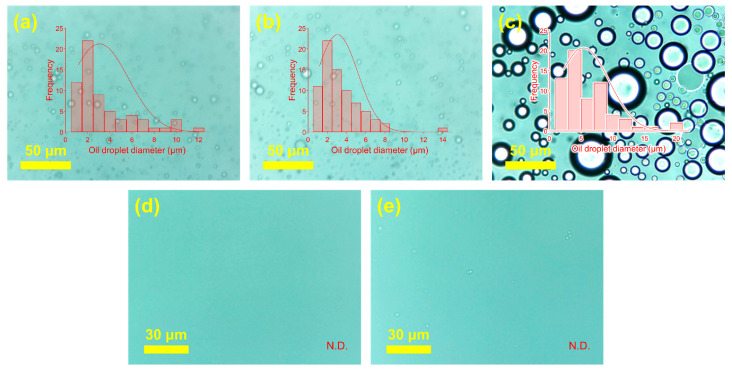
Microscopic images and droplet size analysis of oil emulsion feed and permeate samples: (**a**–**c**) oil emulsion feeds at pH 3, 6, and 12, respectively; (**d**) permeate from the Ch/PES membrane at pH 3; (**e**) permeate from the Ch/PES membrane at pH 12.

Oil emulsion separation tests were conducted under gravity-driven conditions without external pressure. Oil concentrations in feed and permeate were quantified using UV–vis spectrophotometry. The droplet size of the feed oil emulsion varied significantly with pH, measuring approximately 3 µm at pH 3 and pH 6, and 5 µm at pH 12, as shown in Figure 8a–c. Corresponding permeate images further illustrate the separation efficacy of the Ch/PES membrane: at pH 3, the permeate was free of oil droplets (Figure 8d), demonstrating highly efficient oil removal. In contrast, at pH 12, the permeate contained a noticeable amount of oil emulsion droplets around 1 μm (Figure 8e), indicating reduced separation performance under alkaline conditions.

**Figure 9:**
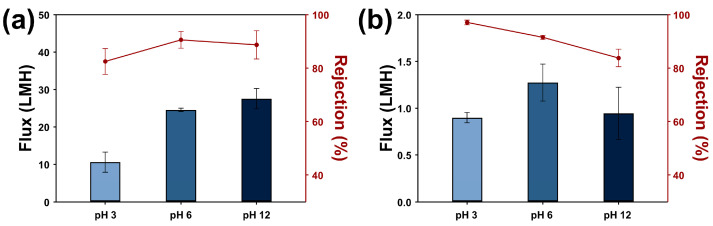
Flux measurements and oil rejection efficiencies of (**a**) PES and (**b**) Ch/PES membranes during emulsified-oil separation tests at varying pH levels.

For the unmodified PES membrane, a significant flux decline was observed under acidic conditions (pH 3, Figure 9a). This is attributed to the pH-dependent stability of the oil emulsions: surfactant-stabilized emulsions produce smaller droplet sizes at lower pH, resulting in a higher concentration of fine droplets that more readily block membrane pores, thereby reducing permeate flux. Moreover, the smaller droplet size at pH 3 likely contributes to decreased oil rejection efficiency.

In contrast, the Ch/PES membrane also showed flux reduction at pH 3 (Figure 9b); however, oil rejection efficiency exceeded 97% under these acidic conditions. This enhanced performance is likely due to the increased hydrophilicity and swelling of the Ch layer, which reduces effective pore size and improves separation of fine droplets. Under alkaline conditions (pH 12), the oil rejection efficiency of the Ch/PES membrane significantly decreased, a behavior attributed to the increased oleophilicity of the Ch coating in basic environments.

### 3.3. Mechanism of Oil/Water Separation Using the Ch/PES Membrane

**Figure 10:**
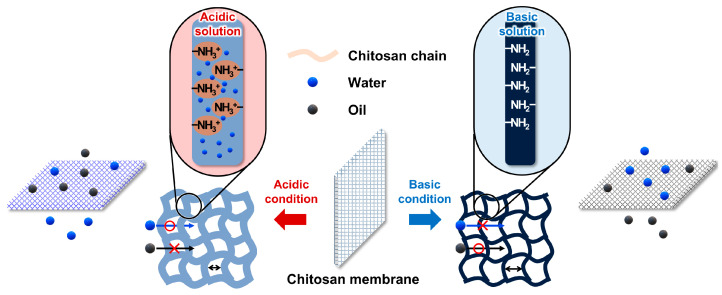
Schematic diagram of the pH-responsive swelling behavior of the Ch/PES membrane.

The pH-responsive separation behavior of the Ch/PES membrane originates from the unique physicochemical properties of Ch, especially the reversible protonation and deprotonation of its amino groups under different pH conditions (Figure 10). In acidic environments, the amino groups of Ch are protonated to form -NH_3_^+^, resulting in electrostatic repulsion between polymer chains. This repulsion induces swelling of the hydrogel matrix due to increased water uptake, leading to an expansion of Ch chains and a consequent reduction in the effective pore volume as the interchain spaces narrow. Under these conditions, the membrane surface becomes more hydrophilic, forming a hydration layer that enhances water permeation while simultaneously inhibiting oil transport. This facilitates efficient separation of oil-in-water emulsions, achieving a rejection rate exceeding 97% in this study.

On the other hand, under alkaline conditions, deprotonation of the amino groups diminishes electrostatic repulsion, allowing polymer chains to pack more closely and aggregate. This results in contraction of the hydrogel network and an increase in effective pore size. The decreased surface hydrophilicity under these conditions enhances oil permeation while suppressing water transport, making the membrane more suitable for the separation of water-in-oil emulsions [41,42,43]. Despite this transition toward oleophilicity, partial separation of oil-in-water emulsions was still observed under alkaline conditions, primarily due to the size exclusion effect of the membrane pores [44].

### 3.4. Implications of the pH-Responsive Ch/PES Membrane

The pH-responsive Ch/PES membrane fabricated in this study demonstrated remarkably variable oil rejection efficiencies depending on the environmental pH. Under acidic conditions, the membrane achieved an oil removal efficiency exceeding 97%, primarily due to increased surface hydrophilicity. In contrast, the efficiency dropped to below 85% under alkaline conditions, attributed to a reversible transition toward hydrophobic surface characteristics. These results demonstrate that the Ch/PES membrane possesses tunable wettability and separation performance, enabling effective separation of oil-in-water emulsions. Based on its pH-responsive surface behavior, the membrane also holds potential for water-in-oil emulsion separation under alkaline conditions. This dual functionality makes the Ch/PES membrane a promising candidate for a wide range of industrial applications, including petrochemical processing, pharmaceutical production, and textile wastewater treatment.

Beyond oil–water separation, the pH-responsive behavior of the membrane offers potential for use in controlled drug delivery systems. pH-responsive membranes have been investigated for biomedical applications in recent studies, indicating that the Ch/PES membrane may also be adapted for specific biological environments if the pH response is appropriately tuned. The membrane’s ability to alter pore structure and permeability in response to pH changes allows for targeted release of therapeutic agents in specific tissues or organs exhibiting distinct pH environments (e.g., tumor microenvironments) [45]. Moreover, the adjustable pore size under varying pH conditions enables the membrane to selectively separate diverse components such as heavy metals, whey proteins, and organic micropollutants [46,47,48]. Therefore, the pH-responsive Ch/PES membrane developed herein holds considerable promise for multifunctional applications in environmental remediation, biomedical engineering, and advanced separation technologies.

## 4. Conclusions

This study developed a pH-responsive Ch/PES membrane for the efficient separation of oil-in-water emulsions under different pH conditions (3, 6, and 12). The successful coating of chitosan onto the PES membrane was confirmed by FTIR analysis, which showed characteristic peaks corresponding to the hydroxyl and amino functional groups. SEM imaging revealed a reduction in pore size of the Ch/PES membrane relative to the unmodified PES membrane, with pore size variations dependent on pH. Porosity measurements indicated that chitosan swelling under acidic conditions increased membrane porosity. Zeta potential analysis demonstrated clear pH-responsive surface charge behavior: positive values below pH 6 and negative values above pH 6, consistently higher than those of the bare PES membrane. UWOA measurements confirmed the oleophobicity of the Ch/PES membrane in acidic and neutral environments, as well as its oleophilicity under alkaline conditions. Oil separation tests demonstrated that the Ch/PES membrane achieved over 97% removal efficiency at acidic pH, attributed to the swelling of chitosan and its enhanced hydrophilicity. However, separation efficiency declined at basic pH due to increased oleophilicity. Overall, this work highlights the potential of the pH-responsive Ch/PES membrane as a versatile and practical material for treating oily wastewater across diverse environmental conditions. Further studies involving various types of oils, such as lubricating oil and industrial waste oil, are necessary to validate the membrane’s performance under realistic oily wastewater conditions. In addition, future work will focus on evaluating the durability and oil rejection efficiency of the membrane over multiple filtration cycles to assess its long-term reusability.

## Data Availability

Data will be made available on request.

## Abbreviations

The following abbreviations are used in this manuscript:

Ch/PES	Chitosan-coated polyethersulfone
FTIR	Fourier-transform infrared spectroscopy
SEM	Scanning electron microscope
SDS	Sodium dodecyl sulfate
MF	Microfiltration
UF	Ultrafiltration
MW	Molecular weight
GA	Glutaraldehyde
HAc	Acetic acid
IPA	Isopropyl alcohol
NaOH	Sodium hydroxide beads
HCl	Hydrochloric acid
DI	Deionized
OCA	Oil contact angle

## Appendix A

**Table A1:** Properties of the oil used in this study.

Type	Viscosity (mPa·s)	Density (g/m^3^)
Canola oil	46	0.920
